# Transformation and time-out: The role of alcohol in identity construction among Scottish women in early midlife

**DOI:** 10.1016/j.drugpo.2014.12.006

**Published:** 2015-05

**Authors:** Carol Emslie, Kate Hunt, Antonia Lyons

**Affiliations:** a Institute for Applied Health Research/School of Health and Life Sciences, Room M410, George Moore Building, Glasgow Caledonian University, Cowcaddens Road, Glasgow, Scotland G4 0BA, United Kingdom; b MRC/CSO Social & Public Health Sciences Unit, University of Glasgow, Top Floor, 200 Renfield Street, Glasgow, Scotland G2 3QB, United Kingdom; c School of Psychology, Massey University, PO Box 756, Wellington, New Zealand

**Keywords:** Alcohol consumption, Gender, Health behaviour, Femininities, Lifecourse

## Abstract

Despite the increase in drinking by women in early midlife, little alcohol research has focused on this group. We explore how alcohol is associated with the construction of gender identities among women aged 30–50 years in the west of Scotland, United Kingdom. We draw on qualitative data from 11 focus groups (five all-female, six mixed-sex) with pre-existing groups of friends and work colleagues in which women and men discuss their drinking behaviours. Analysis demonstrated how alcohol represented a time and space away from paid and unpaid work for women in a range of domestic circumstances, allowing them to relax and unwind. While women used alcohol to construct a range of identities, traditional notions of femininity remained salient (e.g. attention to appearance, drinking ‘girly’ drinks). Drinking enabled women to assert their identity beyond the roles and responsibilities often associated with being a woman in early midlife. For example, some respondents with young children described the transformative effects of excessive drinking which allowed them to return temporarily to a younger, carefree version of themselves. Thus, our data suggest that women's drinking in early midlife revolves around notions of ‘idealised’ femininity but simultaneously represents a way of achieving ‘time out’ from traditional female responsibilities such as caring for others. We consider these findings within a broader social and cultural context including alcohol marketing, domestic roles and motherhood and their implications for health promotion.

## Highlights

• We used focus groups to explore the social context of drinking in mid-life women.
• Drinking alcohol is inextricably linked to gendered identity.
• Women's narratives suggest alcohol facilitates ‘time-out’ from responsibilities.
• Excessive drinking re-connected some mothers with their younger, carefree selves.
• These connections have been noted and exploited by the alcohol industry.

## Introduction

Although men remain more likely than women to drink heavily and experience problems related to alcohol (Emslie & Mitchell, 2009; Plant, 2008), there is growing concern about how women's drinking is changing. A recent systematic review (Smith & Foxcroft, 2009) concluded that an increase in drinking among women is one of the most important trends in alcohol consumption in the United Kingdom over the last 30 years. While the focus of concern is often on younger people, Smith and Foxcroft highlight the increase in drinking that has occurred among older age groups. Indeed, in 2012 (ONS, 2013), similar proportions of women aged 16–24 years, 25–44 years and 45–64 years reported exceeding the UK Government's daily drinking benchmark (3 units for women) on a single day in the previous week (30%, 29% and 30% respectively). This suggests that there is a need for more research on the experiences of female drinkers, particularly those in midlife.

The consumption of alcohol is linked to gender at many levels (Emslie & Mitchell, 2009; McCartney, Mahmood, Leyland, Batty, & Hunt, 2011; Wilsnack & Wilsnack, 1992); qualitative research has demonstrated that the ways in which certain drinking behaviours are adopted, rejected or experimented with can powerfully challenge or endorse ‘idealised’ masculine and feminine identities (Lyons, 2009; Saltonstall, 1993). Gender here is understood as a ‘performance’ that may change according to the context; in sociological terms, it has been argued that ‘doing’ gender involves “creating differences between … women and men… that are not natural, essential, or biological” (West & Zimmerman, 1987, p. 137). In this way, powerful groups legitimise and reproduce social relations that result in or reinforce their dominance so that these gendered distinctions appear natural and normal. In order to respond effectively to changing trends in alcohol consumption, we need a more detailed understanding of the ways in which alcohol is consumed (i.e. whether, when, with whom, and what) and how health behaviours such as drinking can be a powerful and commonplace way through which women (and men) perform a range of gendered identities.

Traditionally, ‘drinking like a man’ – excessive, public consumption of alcohol while simultaneously retaining control over one's body (Lemle & Mishkind, 1989) – served to demonstrate characteristics associated with culturally dominant (i.e. ‘hegemonic’) forms of masculinity such as strength, competitiveness and self-control, and legitimate a hierarchical relationship with femininity and other less powerful configurations of masculinity (Connell, 1995). Women who take on ‘masculine’ characteristics (e.g. authority, lack of compliance) risk being defined as deviant (e.g. ‘sluts’ or ‘bitches); these ‘pariah femininities’ (Schippers, 2007) are feared as they contaminate the relationship between masculinity and femininity. Thus, female drinkers have traditionally been criticised for neglecting their roles as wives and mothers and have been portrayed as sexually promiscuous and lacking in characteristics associated with ‘femininity’ (e.g. being caring, concerned about appearance and health-conscious) (Day, Gough, & McFadden, 2004; Jackson & Tinkler, 2007; Thom, 1997).

Recent qualitative research has found that young women perceive heavy drinking as pleasurable, sociable and socially expected and employ stories about drunken nights out (which sometimes include passing out and vomiting) to facilitate group bonding (Griffin, Bengry-Howell, Hackley, Mistral, & Szmigin, 2009; Guise & Gill, 2007; Lyons & Willott, 2008; Niland, Lyons, Goodwin, & Hutton, 2013; Sheehan & Ridge, 2001; Smith & Berger, 2010; Tutenges & Sandberg, 2013). Peralta (2008) found that young women in the United States used alcohol to excuse ‘gender inappropriate’ behaviour such as taking risks, not worrying about their appearance, and being more assertive in their pursuit of potential sexual partners. Hartley, Wight, and Hunt (2014) reported similar findings for teenage girls in Scotland. At the same time, there have been rapid changes in the social context of drinking in the UK; the liberalization of licensing hours, economic deregulation of the drinks industry and the increased affordability and availability of alcohol has been accompanied by the increasing feminisation of the night time economy and the sophisticated marketing of brands of alcohol to women which explicitly draw on sexual stereotypes (e.g. the ‘Lambrini Girls just want to have fun’ campaign) (Galloway, Forsyth, & Shewan, 2007; Hastings, 2010; Measham & Brain, 2005).

While this may suggest that young women have to some extent appropriated hegemonic masculine behaviours in relation to alcohol, there remain important boundaries that both women and men must negotiate in order to ‘do’ gender appropriately. Contemporary research suggests that drinking beer – particularly out of pint glasses – remains linked to masculinity; that many young women continue to ‘feminize’ what, and how, they drink; and that public drunkenness by other women, particularly older women or working-class women, is still positioned by young women as deviant and embarrassing (Lyons & Willott, 2008; Rúdólfsdóttir & Morgan, 2009). Griffin, Szmigin, Bengry-Howell, Hackley, and Mistral (2013) outline the profound dilemmas and contradictions of femininity for young women in the UK engaging in the current ‘culture of intoxication’; they are supposedly ‘empowered’ and considered independent through drinking excessively, but simultaneously are not supposed to lose control or act in a way considered ‘slutty’. Similarly, Young, Morales, McCabe, Boyd, and D’Arcy (2005) found that excessive drinking had complex connotations for female undergraduates in the United States: ‘drinking like a guy’ did not equate to power or equality but instead emphasised their (hetero)sexuality to male peers. Thus, although more recent cultural portrayals of women's drinking now encompass their increased access to, and consumption of, alcohol, men's drinking is still constructed as ‘different’ to women's (Lyons, Dalton, & Hoy, 2006) and double standards persist (de Visser & McDonnell, 2012).

Very little qualitative research on the social context of drinking has specifically focused on women in early midlife (defined here as 30–50 years). Often, studies either include women at this stage of the lifecourse as part of their sample but do not focus on gender (Ling et al., 2012; van Wersch & Walker, 2009) or concentrate on particular groups of women, such as those with drinking problems (Staddon, 2009) or mothers (Killingsworth, 2006; Waterson, 2000). Rolfe, Orford, and Dalton (2009) interviewed female English ‘heavy’ drinkers (consuming at least 35 units of alcohol in a typical week) aged between 28 and 56 years and their findings provide some useful context for our research. First, they found that these women constructed alcohol as a form of self-medication which altered their mood and helped them to cope and function better in their roles as mothers, carers and paid workers. Secondly, they associated alcohol with leisure and pleasure, and as ‘time out’ from paid work. Thirdly, in order to resist being positioned as unwomanly, sexually promiscuous or lacking in respectability, these female ‘heavy’ drinkers had to perform discursive work such as contrasting the unacceptable public drunkenness of other (frequently younger) women with their own controlled, mainly home-based drinking.

Our study used a qualitative approach to explore how women in early midlife represented their alcohol consumption in the west of Scotland. We have previously reported (Emslie, Hunt, & Lyons, 2012; Lyons, Emslie, & Hunt, 2014) how these women and men of similar ages (Emslie, Hunt, & Lyons, 2013) constructed themselves as experienced drinkers who, through accumulated knowledge of their bodies, could achieve a desired level of intoxication, and placed their parenting and paid work responsibilities at the centre of their drinking practices. However, further analysis showed that this self-presentation of ‘older and wiser’ drinkers was undermined by drinking stories, accounts of peer pressure to drink, and descriptions of using alcohol both as a way to survive the tensions of, and a reward for completing, paid and unpaid work. Here, we focus solely on the female respondents to explore how alcohol is associated with ‘doing’ femininity in early midlife.

## Methods

The DrAM (Drinking Attitudes in Midlife) study aimed to explore the social context of drinking in ‘early midlife’ adults. We were interested in this period of the lifecourse because the alcohol research agenda has focused almost exclusively on younger drinkers (teenagers and those in their twenties). Rather than viewing ‘midlife’ as a singular category, we conceptualise it as socially constructed, fluid and “essentially interactive between people and their environment” (Backett & Davison, 1995, p. 630). Thus, individuals may share some common experiences of changing adult roles and responsibilities and ageing bodies, but are likely to attach different meanings to the experience of ‘midlife’ (Wray, 2007). While we were interested in this broadly defined stage of the lifecourse rather than age per se, for recruitment purposes we focused on adults aged around 30–50 years. Our intention was to recruit a diverse sample. Because we interviewed ‘naturally occurring’ groups (i.e. individuals who already knew each other – see below), this sometimes involved respondents at a similar life stage but of different chronological ages (e.g. one group of mothers of young children ranged from 30 to 41 years).

Qualitative research enables insight into people's (often contradictory) meanings and experiences and also highlights relevant social processes (Chamberlain & Murray, 2008). As we were interested in the social nature of drinking, we conducted focus group discussions with people who knew each other and so could draw on shared narratives and experiences (Kitzinger, 1994). Previous work exploring young people's perceptions of alcohol has used these methods successfully (Lyons & Willott, 2008). We conducted same and mixed-sex groups to accommodate participants’ preferences and to provide greater diversity in the context of the discussions. In this analysis, we focus on the female respondents to explore how alcohol is associated with women's gender identities in early midlife.

Following approval from Glasgow University's Faculty of Law, Business & Social Sciences Ethics Committee, we adopted a broad recruitment strategy: we approached potential respondents on the street and in bars, emailed people inviting them to forward study information to friends and colleagues; approached community groups and workplaces, and advertised on community websites. People interested in taking part were asked to invite up to five friends or colleagues who ‘regularly’ drank alcohol to join them in a group discussion with a researcher, bearing in mind the age group we were interested in (30–50 years). Respondents gave written informed consent to participate and be audiotaped, and completed a drinking grid estimating their alcohol consumption in the previous week. A semi-structured topic guide included changes in drinking over time, occasions when respondents had drunk more than they intended, reasons for any attempts to reduce drinking and distinctions between men's and women's drinking. Respondents were given £20 gift vouchers towards any costs of taking part in the study. Group discussions lasted between 60 and 95 min, were transcribed verbatim and checked against the audio-recordings for accuracy. Pseudonyms were used and identifying features removed. Detailed fieldnotes were written after each focus group discussion and shared with the research team.

Here, we present data from 11 discussion groups (6 mixed-sex and 5 single-sex); details are provided in Table 1. The 34 female participants were all white and came from diverse socio-economic backgrounds. Ten women did not have children; of these, 5 were single with few domestic responsibilities, while 5 were ‘settled’ with partners. Twenty four respondents were mothers; of these, 13 women had young children aged under five years living with them while 11 had older children. Eighteen women reported drinking within the recommended weekly limit (14 units or fewer), while 12 were classed as ‘hazardous’ drinkers (15–34 units) and four as ‘harmful’ drinkers (over 35 units) (Department of Health, 2007; Royal College of Psychiatrists, 2001) on the basis of their self-reported consumption in the previous week.

This inductive study emphasised exploration and theory building. Transcripts were read repeatedly and discussed between the authors. Our analysis was informed by a social constructionist epistemology, which views the world as having multiple systems of understanding that occur through social and cultural experiences, which in turn are largely influenced by language (Burr, 2003; Gergen, 1999). Thematic analysis allowed us to identify, analyse and report patterns in the data, and thus provided rich, detailed and complex accounts informed by our theoretical frameworks (Braun & Clarke, 2006). Themes, sub-themes, and relationships between themes were tentatively suggested, discussed, confirmed, discarded or reformulated with reference to the transcripts in a cyclical process, facilitated by the software package QSR Nvivo. The independent identification of similar themes and patterns in the transcripts by the authors reassured us of the robust and credible nature of our analytic processes.

## Findings

### Overview

These women in early midlife described how they associated drinking alcohol with relaxation and temporary escape from the obligations of paid work, domestic work and/or striving to meet the needs of others. Below, we describe two main themes which emerged from our data which interconnect in complex ways. First, even though these women used alcohol to perform many different versions of ‘femininity’, the continuing salience of traditional notions of femininity (e.g. in relation to physical appearance and consuming appropriately ‘feminine’ drinks) was notable as a constant background to their discussions. Secondly, some respondents used alcohol to assert their identity beyond the roles and responsibilities associated with being a woman in midlife. We argue that women's drinking at midlife remains tied to the performance of ‘idealised’ femininity but *simultaneously* represents a way of achieving rapid and convenient ‘time out’ from traditional female responsibilities that often characterise this life stage, particularly caring for others.

#### (1) Using alcohol in performances of gender in early midlife: ‘girly girls’ to ‘playing the lad’

Women discussed consuming different alcoholic drinks according to their mood, the season, the time of day, the price, where they were drinking, their companions and the formality or function of the occasion. For example, cocktails, (fizzy) wine and Pimms were described as appropriate drinks when ‘with the girls’ (FG15: ‘cocktails and fizz… you’re all dressed up with the girls’; FG8: ‘out with the girls… you get cocktails and you’re so excited that they’re colourful & fizzy… hoofing wine, laughing your head off!’; FG4: ‘with a crowd of girls… everybody brings a bottle of wine and it is rowdy and riotous!’). The extract below from FG12 demonstrates how different drinks were matched to gendered identities (from cocktails at one end of the spectrum to pints of beer at the other) and how clothing and drinks were deployed for different performances of gender on different occasions (high heels and wine vs. trainers and beer):

LYNN: A little beer to start off the night… or if I’m feeling maybe a bit more grown up, I’d have a glass of wine. If I’m in the mood for a big night out, DEFINITELY Southern Comfort!

RUTH: I agree it depends how you feel but I think it also depends who you’re with … If I was out with you … shopping and we went into a bar we would have like a cocktail just to be girly…

ERIN: Depends what you’re wearing: if you’re wearing your trainers it's a beer, (if) you’re wearing your high shoes it might be a glass of wine. [laughter]! …

LYNN: If I was going to a party… dressed up, I don’t think I would go for a beer. Like I wouldn’t have a pint or anything like that, I think I would have some kind of a smaller glass or a wine or something like that.

RUTH: I was at a wedding just the end of last year and I sat with a bottle of beer …I just don’t care anymore …(if) people want to go “Oh look she doesn’t look very girly”, I’m just like ‘well, you know, I don’t care’.

ERIN: I think a bottle of beer, though, does look more girly than a pint.

RUTH: Oh definitely, than a pint, definitely….

ERIN: There's probably part of my brain that's still my dad saying [whisper] ‘Girls don’t drink out of pint glasses’ and just giving me a dirty look … I think if you’re sitting with your jeans and … a big pint that's great. but if it's in a nice party dress it does look a bit incongruous to me. [laughter] (FG12)

This extract illustrates respondents’ awareness of the continuing social pressures to drink in a ‘ladylike’ way (‘she doesn’t look very girly’, ‘girls don’t drink out of pint glasses’) and the subtleties of gendered identity and drinking receptacles (more ‘girly’ bottles compared to ‘big pints’ of beer). It also reveals the continuing importance of clothes and appearance for these women in midlife; accounts from those with and without children, from both affluent and deprived areas, illustrate that they are very aware of ‘how they look’ – and how they appear to others – while consuming different alcoholic drinks. Appearance was also salient when drinking with female friends in domestic settings (e.g. FG8: “The mums… sit down minus children, husband, dishwashers and have a drink and a chat. We get dressed for it. We actively get dressed, get changed, hair done, make-up on”).

Women spontaneously discussed their own consumption of beer in half of the focus groups (four single sex and two mixed sex). However, women's consumption of beer from pint glasses was a much more contentious issue. Madeleine (FG15), a professional woman with a young child, was forthright about discussing her love of drinking pints of beer (and real ale), and explicitly linked this to her performances of gender (‘playing the lad’) when with male colleagues in a work context. She was unusual in being the only woman in the sample who was entirely unapologetic about currently drinking in this ‘masculinised’ way, although her account echoed other women's ‘pride’ at being able to ‘handle their drink’ and ‘keep up with the banter’ in mixed company:

MADELEINE: When you’re out … (with)…a bunch of blokes (in a work context), sometimes you feel like they’re kind of watching you …as you have your first or your second pint and somehow I … feel like there's a little pride thing … not to the point where you sort of like try and drink them under the table!…. I quite like sort of surprising people a little bit, showing that you can kind of keep, keep up. (laughter) …I’d be MORE likely to have a pint if I’m out with a bunch of blokes who are all having pints but I could equally have a glass of wine. But I suppose there's maybe part of me …that thinks I’m kind of playing the lad a little bit, you know… not very convincingly…in a girly-girly kind of way! (FG15)

Madeleine's senior job in a public sector organisation may perhaps explain her ability to openly position herself as drinking in this ‘masculinised’ way; because of her class position she did not need to publicly ‘invest’ in femininity as she had alternative resources to draw upon to construct herself as (respectably) feminine (Skeggs, 1997).

Within the single sex groups, women often expressed different opinions about drinking pints. They resolved these disputes through relegating drinking pints to a previous stage of the lifecourse (e.g. youth, student days) or to particular occasions (informal occasions or one-off events). For example, in FG8, Isobel stated categorically that she couldn’t ‘stand seeing women drinking pints’, linking this to crude, loud, inappropriate female behaviour (‘ladette’), which Vicky (younger than Isobel, living in a less affluent area and employed by Isobel) hesitantly challenged. The group worked hard to resolve this potential breach of ‘idealised’ femininity by suggesting drinking pints was acceptable on certain occasions (a ‘once a year blow out’ rather than as a routine occurrence):

ISOBEL: I’ve always had a thing about the ladette behaviour. I can’t stand seeing women drinking pints, it's just a real no-no for me. Or drinking out a bottle – I don’t care if it's trendy beer or lager … I just don’t think it looks right. Tattoos, loud behaviour, too much cleavage.

VICKY: See that’ll be personal – cause I’ll still do that a little bit, if it's in the right situation. If I’m at a gig, if I’m at T in the Park (music festival sponsored by a Scottish brewery), I’ll be drinking out of a pint glass, but I wouldn’t do it as much now.

HANNAH: Not in a busy city centre street or…

ISOBEL: No, I was gonna say – I’ve never seen you drink or behave the way I’m talking in a social – you’re talking about your once a year blow out.

VICKY: Right, OK, yeah. (FG8)

Respondents in the mixed sex groups did not (openly) disagree with each other on their views about women drinking pints of lager or beer. One man in FG3 commented briefly that ‘a lot of females drink pints… you don’t really notice that’ and then changed the subject. In contrast, the other two mixed sex groups who discussed this (FG1 and FG6) were negative about women drinking pints under any circumstances. For example, Andy in FG1 – encouraged by Craig – described female pint drinkers in strongly negative terms (‘horrendous’, ‘utterly bizarre’) and Debbie backed them up, suggesting that female pint drinkers were ‘butch’ (i.e. unfeminine and possibly lesbian).

Thus, respondents’ narratives suggested that an awareness about what they drank, how they looked and how these ‘props’ (i.e. beverage, receptacle, clothes and accessories) linked to appropriate performances of femininity was common to most women in the sample in early midlife, regardless of domestic circumstances or parental and social status.

#### (2) ‘Time out’ and ‘transformation’ across the lifecourse

Our sample was intended to reflect the social and lifecourse diversity of women in early midlife, and this was clearly illustrated by the range of different living arrangements (e.g. 34 year old woman living with her partner, 44 year old single mother living with three children aged 11–17, 45 year old woman living with her partner and toddler, 47 year old woman living with her partner and 20 year old daughter, 50 year old single women without children living alone). As described in our previous work (Emslie et al., 2012), the respondents in our study recognised three lifecourse stages linked to different expectations of drinking (see also Backett & Davison, 1995). First, ‘singles… with no responsibilities’ (Isobel, FG8) had the freedom to go straight to the pub from work and had the time and opportunity to recover from hangovers in bed at the weekends if necessary. Secondly, those who has ‘settled down’ as a couple but did not have children had fewer opportunities for socialising due to more demanding jobs, financial responsibilities and longer commutes. Thirdly, respondents with children at home were perceived to have more limits on their drinking, although these limits changed as children grew up and became more independent. The themes that emerged from our data (‘time out’ and ‘transformation’) were linked to these different lifecourse stages.

Five women in the sample did not have children, were not in ‘settled’ domestic routines with partners and presented themselves as having relatively few obligations apart from paid work. Their descriptions of (often excessive) drinking involved fun, sociability and bonding with friends or partners. For example, Rach and Grace (50 and 49 years old respectively, both ‘hazardous’ drinkers, FG5) described frequent socialising with sales colleagues who want to keep drinking all night (‘out to the death’) while Tara (31 years old, ‘harmful drinker, FG11), described drinking ‘more in the last three months than I have in the last two years’ as a result of meeting a new partner. These descriptions had much in common with the accounts of young people reported elsewhere (Griffin et al., 2009; Szmigin et al., 2008) and illustrate how traditional transitions into adulthood have become increasingly elongated and diverse (Furlong & Cartmel, 2007).

The remainder of this section of the paper excludes these five women. It focuses on the remaining 29 women for whom alcohol represented a way to achieve ‘time out’ from paid work and domestic responsibilities. This played out differently according to living arrangements. The accounts of five women without children and 11 women with older children suggested that alcohol represented valuable ‘time out’ from busy lives. However, the accounts from the 13 mothers of young children (aged under 5 years) suggested that, while alcohol helped them to achieve ‘time out’, it also performed a transformative function. We describe these two groups in turn.

**(a) ‘Time out’ from responsibilities (‘settled’ couples and mothers with older children)**

For most women in the sample, drinking was associated with an embodied sense of enjoyment which helped them to relax, and represented a time and space away from paid work and domestic responsibilities (Lyons et al., 2014). This was described differently by women in settled domestic routines without children, compared to women with older children. The former group described the importance of setting aside time to focus on their partners and their accounts suggested the importance of alcohol to demarcate this time and space. Respondents referred to this as the ‘lovey stage’ (Grace FG5) when a ‘new-ish couple’ would want to have ‘that lovely meal and … nice bottle of wine with it’ (Eleanor, FG3). Respondents discussed the importance of regularly eating and drinking (usually wine) with their partner, marking out time and space away from the demands of domestic and paid work. Hannah describes this as ‘an hour… of niceness’, while Mandy emphasises how she looks forward to opening a ‘nice bottle of wine’ with her partner and how she would ‘never want to… cut that out’:

HANNAH: The main focus of our evening is our main meal in the evening, and we always have wine with our meal … I like to set the table and sit down …cause it's the only chance you get to sit down and have … an hour sort of niceness, then, before you’ve got to load the dishwasher, do the ironing, you know… So we do that every night. (FG8)

MANDY: We (Mandy and her partner) would often, like, open a bottle of wine on a Friday night, and have the bottle between us, and occasionally we might open a second bottle… One of the things I enjoy in a week is coming home from work on a Friday night and opening a nice bottle of wine, looking at the wine rack, and thinking, “Which one will we open?”, you know? And, like I really enjoy that, so I would never want to…cut that out. (FG10)

Unsurprisingly, mothers of older children had more demands on their time than those without children. Drinking was equated with a release from responsibilities after a hard day at work or juggling paid work and childcare, and presented as having to be fitted around childcare responsibilities. Many of these women described how they had stopped, or reduced, their drinking when their children were young but how they were now able to drink more as their children became more independent. However, respondents emphasised that mothers retained the main responsibility for their children, so they still had to be ‘on duty’, and expressed concerns about the effect on children if they saw their mothers drinking (excessively). Their accounts stressed the importance of making the most of limited ‘me time’. While the ‘time out’ narratives (above) of women in settled domestic routines without children focused on making time for partners, mothers of older children often focused on time to socialise and drink with female friends. Both Isobel and Stella spent some time outlining how their drinking was arranged around work and childcare responsibilities (‘my drinking is controlled by my children… you’re up early in the morning – packed lunches, hangover, school run – it doesn’t work!’; ‘as women, you’re the main carer for your child… you’re still taking all that responsibility on board… in an emergency, somebody has to be sober’), before describing the fun they had with female friends. Isobel states that this is ‘female down time’ where men are not welcome, while Stella suggests that female drinking without men is ‘riotous’ and ‘inhibitions just go’:

ISOBEL: My husband would not cross over to the girls’ night. It's strict division of sexes. The women have a get together … (the men) have to do the children – you can’t join the women. And if you wander into the kitchen when the women are there, the conversation stops and there's a pointed conversation move – get out. You’re not welcome, and the boy is chased out the kitchen because it is the female down time. (FG8)

STELLA: I think when it's women altogether it can get quite riotous, and it's like you know, especially when there's drink involved, your inhibitions just completely go. And there's no men, and the conversation is very uninhibited, it's all about sex and moaning about men. You know so really, and all the faults come out, you know and you’ve not got men there to curtail the situation so it's, anything goes…. (FG4)

Thus, for these mothers of older children, drinking with other women was constructed as facilitating female friendships and providing a physical space away from men where women could be candid and unrestrained (Bachmann, 2014).

**(b) Transformation (mothers of young children)**

The accounts of mothers with children aged under five years of age suggested that the consumption of alcohol could be associated not only with ‘time out’ but also with metaphorical transportation across the lifecourse. Drinking alcohol at home with partners when children were in bed was constructed as creating an ‘adult’ space at home, while (excessive) drinking with friends was associated with an earlier, more carefree stage of life. These women therefore used alcohol to assert their identity beyond the role and responsibilities associated with being mothers of young children.

*(i) Using alcohol as a ‘declaration of adulthood’ with partners*

Given the trouble and expense of organizing childcare to go out for a drink, mothers of young children discussed how drinking wine was a good way to relax at home with partners (“let's bring the pub to our living room!” FG12). Some women therefore used alcohol to demarcate civilised ‘adult’ time, using it as a treat to enjoy with their partner once children were in bed. This has parallels with the narratives of the ‘settled’ couples without children above. However, mothers of young children used alcohol to demarcate ‘civilised’ space away from children within the household, and also to construct themselves as autonomous adults. For example, Anne (FG12) described her first glass of wine on a Friday night as a ‘declaration of adulthood’, a phrase with strong connotations of independence. She went on to explain why, when her children were very young, she waited until they were in bed so she could fully enjoy drinking. Her narrative suggests advantages to a clear distinction between time with her children and ‘adult’ time:

ANNE: (A) bottle of wine on the Friday night and a nice meal would be like a big, big treat … once the first glass goes down, just that nice sense of relaxation that comes with it. … it would be a kind of like, a declaration of adulthood … once they’ve (children) got to bed … Just trying to have a kind of bit of atmosphere with your husband … not just like sitting on the couch and staring at the telly…we’re doing something together. (later) … if I was to have like a drink of wine in the afternoon (at a summer barbeque), I would want another one (agreement), … and then I … just get really resentful of my kids then … I’ve still got to go and give you dinner and give you a bath and do all this other stuff … It just reminds me of the limitations of being a mum. … just protect yourself from that exasperation! (FG12)

Women in FG15 also discussed the rituals of ‘wine o’clock’ or ‘beer o clock’ (the timing of first drink of the day), which functioned as a reward (or ‘me time’) at the end of a busy day. Madeleine was happy to blur the distinctions between ‘child’ and ‘adult’ time by reading bedtime stories while having a drink, although careful to explain how she still had her ‘rules’ about when to start drinking:

MADELEINE: Six o’clock is kind of like well, the evening's starting … you can either come home from work and have a drink or sort of feel like the end of Mummy Day, it's coming to its beautiful end! (much laughter) … I will sometimes be reading stories with a glass in my hand. That's like fine but I think if somehow if I… if I (laughs) it's my little rule, you know, if I let myself like drink at five o’clock I think…ooohh! … (laughter) (FG15)

A number of women told stories about men supporting their partners to resume socialising in contexts which involved drinking – and, by implication, return to their ‘normal’ selves – after having children. For example, one women (FG8) described going out for Sunday lunch at the pub with other mothers while their partners took charge of the children, and how the woman with the youngest baby fell asleep on her handbag without tasting a drop of alcohol. In FG12, Anne's description of her husband's supportive actions (appearing ‘with the (milk) bottle and a glass of red wine’) which signalled the end of her attempts to breastfeed her youngest child, was met with appreciative ‘aahs’ from the rest of the group. This suggests that male partners also perceived alcohol as helping women to cope with the rigours and demands of motherhood:

ANNE: One night, he was like “well I’ll just go and get a bottle ready, and then you know, just see how you get on” … and you know I was all in tears and everything and trying to feed this baby… and then he came upstairs with the bottle and a glass of red wine! (Others - aah!) And I took one look at the glass of red wine and I was like “yeah let's just bottle-feed!” (laughter] … So, yeah, I think it had become like a real thing that I kind of felt like was.

RUTH: Freedom!

ANNE: … yeah, was freedom, and was not being a mum, and was not being responsible and all that kind of stuff. And really kind of welcomed that in that moment. But also, yeah, you know every Friday night (I) kinda welcome that. (FG12)

As Anne's account suggests, drinking was constructed as welcome ‘freedom’ (albeit temporary) from the work of being a mother of young children.

*(ii) Using alcohol to return to a ‘carefree’ youthful self*

Mothers of young children described how (excessive) drinking seemed, in the moment, to return them to their youth. Alcohol thus allowed these women to perform a younger, carefree version of themselves having fun, literally providing ‘time out’ from parental responsibilities but also metaphorically transporting them to an earlier stage in life. For example, women with young children talked about how particular drinks summoned up their youth (FG9: “If I have a long vodka now, I feel about 10, 15 years younger!” FG12: If I’m out …I’ll buy …Bacardi and coke … that's what I used to drink when I was young, free and single!”). This suggests that particular tastes (like smells and music) have nostalgic associations with past good times and earlier constructions of ourselves.

Many of these accounts focused on excessive drinking. For example, Madeleine (FG15) described herself as ‘kiddish’ because she sometimes didn’t pay attention to what or how much she was drinking and so ‘accidentally on purpose’ got drunk; this provided a stark contrast with the detailed planning that habitually characterised her life as a professional woman and a mother:

MADELEINE: It's (drinking) just a kind of normal part of life really. … I rarely…sometimes I will accidentally on purpose, really get drunk but mostly sort of stop before that…. I think I must be really quite kiddish because actually I don’t really think too much so if most of the table are having ‘X’, I might well just have the same and then just kind of see how it goes and then I might be struck by surprise and then suddenly …if people are round at the house… “I don’t really understand why I feel poorly!” (drunk voice) And then I’ll wake up in the morning and I’ll see like you know, about six to eight bottles and I think… well, there was three of us (laughter)! (FG15)

The following extract from FG12 illustrates how excessive drinking could be constructed as a ‘release valve’ which helped women with young children cope with their gendered social roles. Lynn and Ruth explicitly described how their nights out enabled them to ‘regress’ to ‘being teenagers’, escape from their work and domestic responsibilities and remember ‘who you are’. This suggests that even in the 21st century, mothers of young children still struggle to (re)construct and (re)claim identities (“I’m still me”) beyond their roles as mothers, partners and employees:

LYNN: I don’t think I could ever just be a moderate drinker. It's – you binge, you don’t do it again for three months, you binge. And it's just like that, you know.

RUTH: It's like a kind of, like, release valve, like a kind of pressure valve, letting off steam and -

LYNN: There is quite a build up to our nights out …we regress, I think! (laughter). We go back to just being teenagers, really, and we talk about what we’re doing with our hair, and our shoes, we’ll swap dresses …it is like going back to reliving your youth.

RUTH: It is a social side as well, because you don’t get the chance when you have to be responsible, you’ve got work or you know children or both and you’re just like – to go back to that time when you felt free …Escapism. Time for ourselves.

LYNN: After I had (daughter)…she was about eight or nine months before I went out. … I was like ‘no, I’m a mum now, I’m responsible’. …I think I thought I was more grown-up mentally than what I actually am. … I love going out, and I’d missed it when I’d been pregnant so it was a good way to get back to normal… I think it's just – about remembering who you are, rather than being a mum all the time. (agreement) You know, it's – it's hard to remember you were a person before you had babies. [laughter] … I’m still me, I’ve just got two kids to look after now.

RUTH: You’re more than a wife and a mother, you’re yourself as well. (FG12)

Lynn discussed her ‘binge drinking’ in the extract above, and went on to describe how her husband would look after their children for the weekend four times a year to give her the opportunity to go out drinking with her friends with plenty of time to ‘recover’. (It was notable that women in the sample without children commented that friends with young children were more likely to drink heavily on nights out, as they had fewer opportunities to do this.) The extracts from narratives below illustrate how women with young children explicitly referred to ‘binge’ drinking, ‘blow outs’ or getting drunk, linking this to more limited opportunities for drinking and/or going out (‘blue moon’, ‘make the most of it’):

DENISE: Because I’m like a single mum now and I don’t get out. So when I have a once-in-a-blue-moon chance of going out, I’m gonna to go out and I’m gonna to get drunk because I know I can’t any other time. … if I go out I’m out till the death (laughter), I’m out til closing time! (FG15)

JODY: I think I’m in the group that you would call a binge drinker… I’ve just been busy with the baby, and I know when I do have a drink, the next morning… I can’t really cope with it. … But I’ll go so long and then just one day, I think I’m fed up or I’m bored, I’ll get a bottle of wine for when she goes to bed. … I say I’ll have a GLASS of wine, but I never – I’ll just have the bottle. (FG5)

FI: I probably fit more into this classic ‘binge drinker’ now than I did when I was younger, because I drink less often. So it's like make the most of it, we’re out on a Saturday night, you know, grandparents have got the kids till tomorrow lunchtime, we can go home and we can have a long lie (get up late), so I’m not watching, thinking, ‘oh, well, I’m up early, I need to stop now’ (FG10)

Thus, given the constraints on women with young children in early midlife, (excessive) drinking was presented as a way of making the most of limited leisure or ‘me time’. It was also understood as a way of resolving multiple co-existing femininities while keeping a coherent sense of one's self and identity.

## Discussion

In this study, meanings attached to drinking alcohol by women in early midlife were inextricably bound up with gendered identity. Respondents described adopting a range of positions at different times, from ‘girly girl’ to ‘playing the lad’, through their choice of alcohol, drinking vessel, drinking companions and clothes, while distancing themselves from ‘pariah’ femininities (e.g. ‘ladette’, ‘butch’: Schippers, 2007). With the exception of a few respondents without domestic responsibilities, women's lives, and their drinking at this stage of the lifecourse, were centred on ‘idealised’ notions of femininity (e.g. orientation to others’ needs, domestic chores, childcare). Our data suggest that alcohol played an important role in providing ‘time out’ from mundane aspects of day-to-day existence for many women in early midlife. In addition, the accounts of mothers of young children suggested that (excessive) drinking transported them temporarily back to ‘carefree’ youth, reliving a time before the responsibilities of paid work and parenthood. Our study represents an original contribution to the alcohol research literature, which has focused on younger drinkers and ignored this stage of the lifecourse. We conclude that drinking – sometimes excessively – is closely tied to the performance of ‘idealised’ femininity (manifested most obviously in appearance), but simultaneously represents transitory relinquishing of traditional female responsibilities at midlife.

Psychoactive substances have been used across cultures to alter experiences of time. For example, Reith (1999) fascinating work on drug addicts found an almost exclusive focus on the present as respondents gave accounts of ‘lost time’ and time ‘standing still’. However, the gendered nature of substance use in relation to ‘time out’ is rarely discussed. Women have less ‘free’ time than men; they retain responsibility for caring and domestic work as well as ensuring there are smooth transitions between the worlds of home and paid work, and are expected be constantly ‘on call’ for children in a way that fathers are not (Connell, 2005; Deem, 1996; Paradis, 2011). For women in our study, alcohol represented a quick and achievable way to create time away from obligations, often without leaving the house. This liminal period was constructed as a restorative process where time was experienced differently, bodily sensations were intensified and shared stories helped to combine fragmented experiences into unified biographies. This identity work has clear parallels with other research (see Elsrud, 1998). Women often described drinking with friends and loved ones as pleasurable and uplifting, and our previous work has explored the desirable embodied experiences of reaching and sustaining a particular level of intoxication with like-minded people (staying ‘in the zone’, Lyons et al., 2014). In the current paper, we have described how drinking alcohol – whether to create civilised ‘space’ with partners or riotous, hedonistic excess with friends – functioned as a way for women to carve out ‘me time’ and, more specifically, as a way for mothers of young children to reconnect with earlier more ‘carefree’ selves.

It is important to recognise that these respondents were not just describing their drinking practices in these focus groups, they were also actively ‘performing’ gender within the discussions. With few exceptions, women worked hard discursively to position themselves as ‘respectable’ female drinkers (in terms of appearance, control over their behaviour and consuming appropriately ‘feminine’ drinks). They also made it clear that their drinking was age and lifecourse appropriate, and only took place after they had met their responsibilities as employees, partners and/or mothers (Emslie et al., 2012). Respondents drew on contemporary discourses of motherhood where appropriate attention to maternal responsibilities is combined with seeking ‘me time’ where the desire for relaxation and autonomy can be fulfilled. The alcohol industry – following Big Tobacco – has been adroit at (re)creating associations between women's drinking, pleasure and independence (Amos & Haglund, 2000; Lyons et al., 2006; McCartney et al., 2011). For example, the concept of ‘wine o’clock’ as ‘me time’ for busy mothers is explicitly used to market alcohol by MommyJuice wines (“whether it's playdates and homework… or finding that perfect balance between work and home, Moms everywhere deserve a break. So tuck your kids into bed, sit down and have a glass of MommyJuice – because *you* deserve it!”: http://mommyjuicewines.com/).

Alcohol plays a key role in the ‘transformation of the self’ (Bancroft, 2012) but our findings suggest this varies across the lifecourse. While Peralta's (2008) college students asserted that ‘alcohol allows you not to be yourself’; women in early midlife in our study described drinking as a way to carve out ‘me time’ and to (re)connect with ‘who they were’. Thus, in youth, alcohol may allow the trying out of different identities (Hartley et al., 2014), whereas in early midlife, drinking may allow a reconnection with previous – or seemingly more ‘authentic’ – selves. There are parallels with de Botton (2012) argument that because familial roles come to dominate long-term relationships, moving out of the prosaic domestic environment (to a hotel room, for example) may help people reconnect with long lost sexual selves; alcohol similarly allows this temporary ‘movement’ out of daily domestic roles and space.

In particular, the transition to motherhood often involves a reformulation of women's sense of self (Haynes, 2008) and our data suggest that alcohol may have become an important means for some mothers to articulate this identity work. Killingsworth (2006) has argued that alcohol is used as a tool to demonstrate agency when constructing and balancing the ‘fractured’ identities of ‘mother’ of young children (with connotations of mundane physical work, dependence and passivity) and ‘independent woman’. Thus narratives about (excessive) drinking may help mothers of young children to resolve multiple co-existing femininities and keep a coherent sense of self and identity, before a return to gendered responsibilities.

Our study has some limitations. We used friendship discussion groups to explore co-constructed accounts of socialising and drinking. However, the group context may have made it more difficult for women to discuss dissenting viewpoints. Secondly, like other qualitative studies, our respondents were self-selected. Although we advertised for people who drank ‘regularly’, almost half the sample reported drinking over the recommended weekly limit for women of 14 units. Finally, we did not recruit any women who identified as gay or bisexual, which would have provided a more diverse exploration on how alcohol is linked to ‘doing’ gender. It is rare to find work on drinking which includes heterosexual, gay and bisexual respondents and this should be rectified.

Like the young working-class women described by Brown and Gregg (2012), many respondents in our study had limited opportunities for fun and drinking because of their domestic and work responsibilities; drinking thus provided “temporary relief from seemingly fixed selves and relationships at a time when actual opportunities for liberation may be limited” (p. 365). Health promotion efforts need to be sensitive to cultural constructions of gender at this stage of the lifecourse. Future research could explore alternative strategies which women use for demarcated time out and relaxation, which also provide opportunities for reformulating their sense of self (e.g. exercise, socialising without drinking excessively and other ways in which they carve out ‘me’ time). If (excessive) drinking is partly a response to perceived gendered constraints in early midlife, this seems unlikely to change in the short term, given the persistence of the unequal distribution of domestic labour.

## Conflict of interest

None declared.

## Figures and Tables

**Table 1:** Details of focus groups and participants.^a^

	Group type	Participants	Ages	Deprivation category^b^	Alcohol units past week	No. drinking above ‘recommended’ limits^c^	No. parents (with kids <5 years)
1	Council workers	2 M, 2 F	44–49	Intermediate	9–15	1 F	2 M, 2 F (0)
3	Lecturers	2 M, 2 F	34–49	Mixed	21–33	2 M, 2 F	2 M, 1 F (0)
4	Female friends	4 F	44–48	Affluent/intermediate	14–60	3 F	4 F (0)
5	Sales workers	1 M, 4 F	40–50	Mixed	9–36	1 M, 3 F	1 F (1F)
6	Community group (deprived area)	4 M, 3 F	41–mid 50s?^d^	Deprived	0–92	1 M	1 M, 2 F (0)
8	Office workers	4 F	36–47	Affluent/intermediate	0–27	2 F	2 F (0)
9	Community group (affluent area)	3 F	35–45	Affluent	3–15	1 F	2 F (2 F)
10	Heterosexual couples	2 M, 2 F	32–35	Affluent/intermediate	14–28	1 M, 1 F	1 M, 1 F (1 M, 1F)
11	Best friends and girlfriend	2 M, 1 F	31–33	Mixed	25–65	2 M, 1 F	1 M (0)
12	Gym group mothers	4 F	30–31	Deprived	3–20	1 F	4 F (4 F)
15	Toddler group mothers	5 F	30–41	Affluent/intermediate	1–19	1 F	5 F (5 F)

F, female; M, male.

a This dataset excludes groups 2, 13 and 14 which were all male groups and group 7, who were non-drinkers recruited for an alternative perspective on the cultural context of alcohol.

b Carstairs scores calculated for residential postcodes: affluent = DEPCAT 1 and 2, intermediate = DEPCAT 3–5, deprived = DEPCAT 6 and 7, mixed = respondents from each of these three categories present in one group.

c Over 21 units/week for men, 14 units/week for women.

d Two participants in FG6 did not give their age.
